# Supplementary figures and images for: Experimental and mathematical insights on the interactions between poliovirus and a defective interfering genome
Source: PLoS Pathog. 2021 Sep 27;17(9):e1009277. doi: 10.1371/journal.ppat.1009277 (PMC8496841; doi:10.1371/journal.ppat.1009277)

### Dually transfected cell

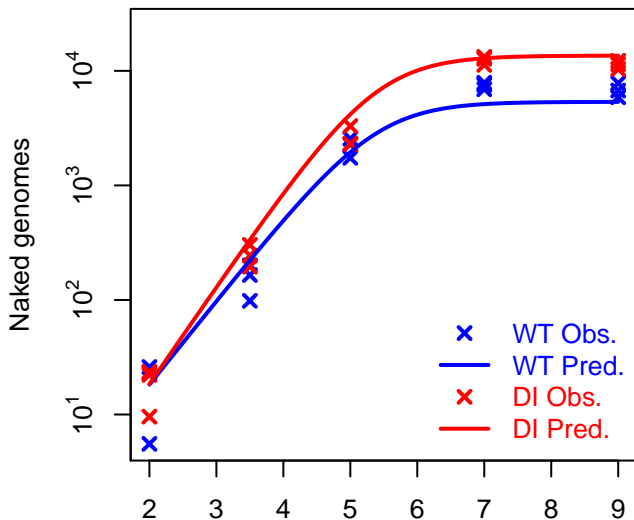

### Singly transfected cell

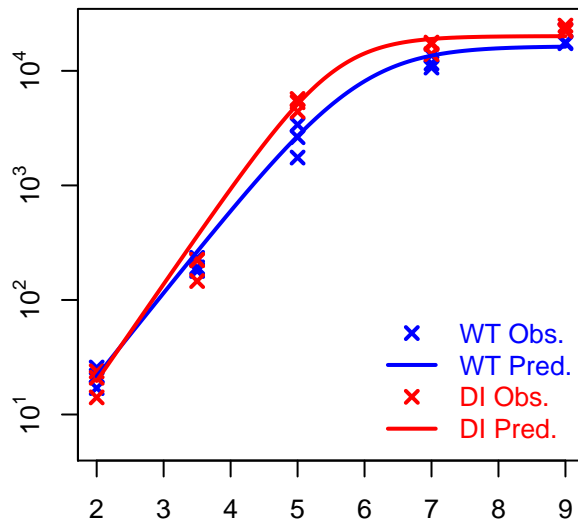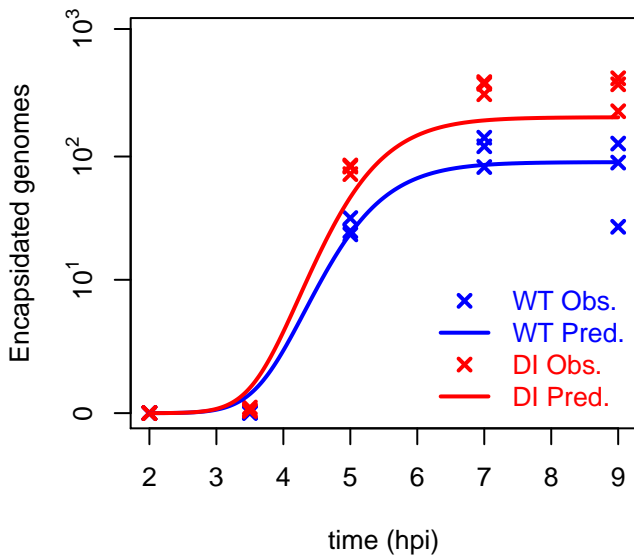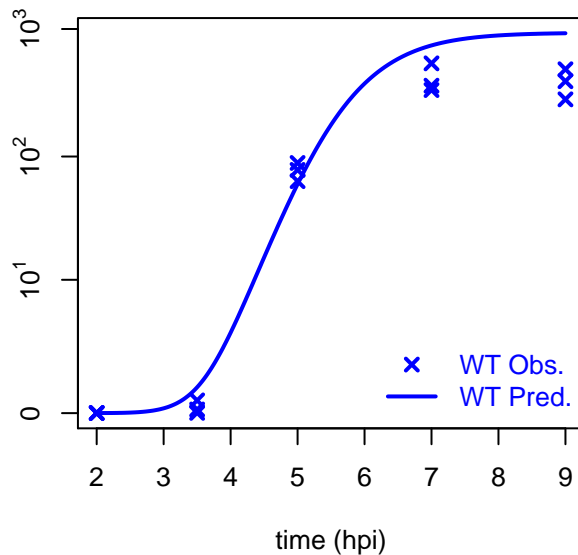

Supplement: S1 Fig — Evolution of the number of WT and DI (A-B) naked genome copies and (C-D) encapsidated genome copies with time, from 2 to 9 hours post transfection (hpt). (A & C) show data in dually transfected cells whereas (B & D) show data in singly transfected cells. WT and DI results are shown in blue and red color, respectively. Crosses indicate experimental data for 3 replicates per sampling time at 2, 3.5, 5, 7 and 9 hpt. Solid curves show the fit of the full model M12 with same encapsidation rate for WT and DI naked genomes (Eqs. (S10)–(S13) in S1 Text). (PDF) [file ppat.1009277.s001.pdf]

A

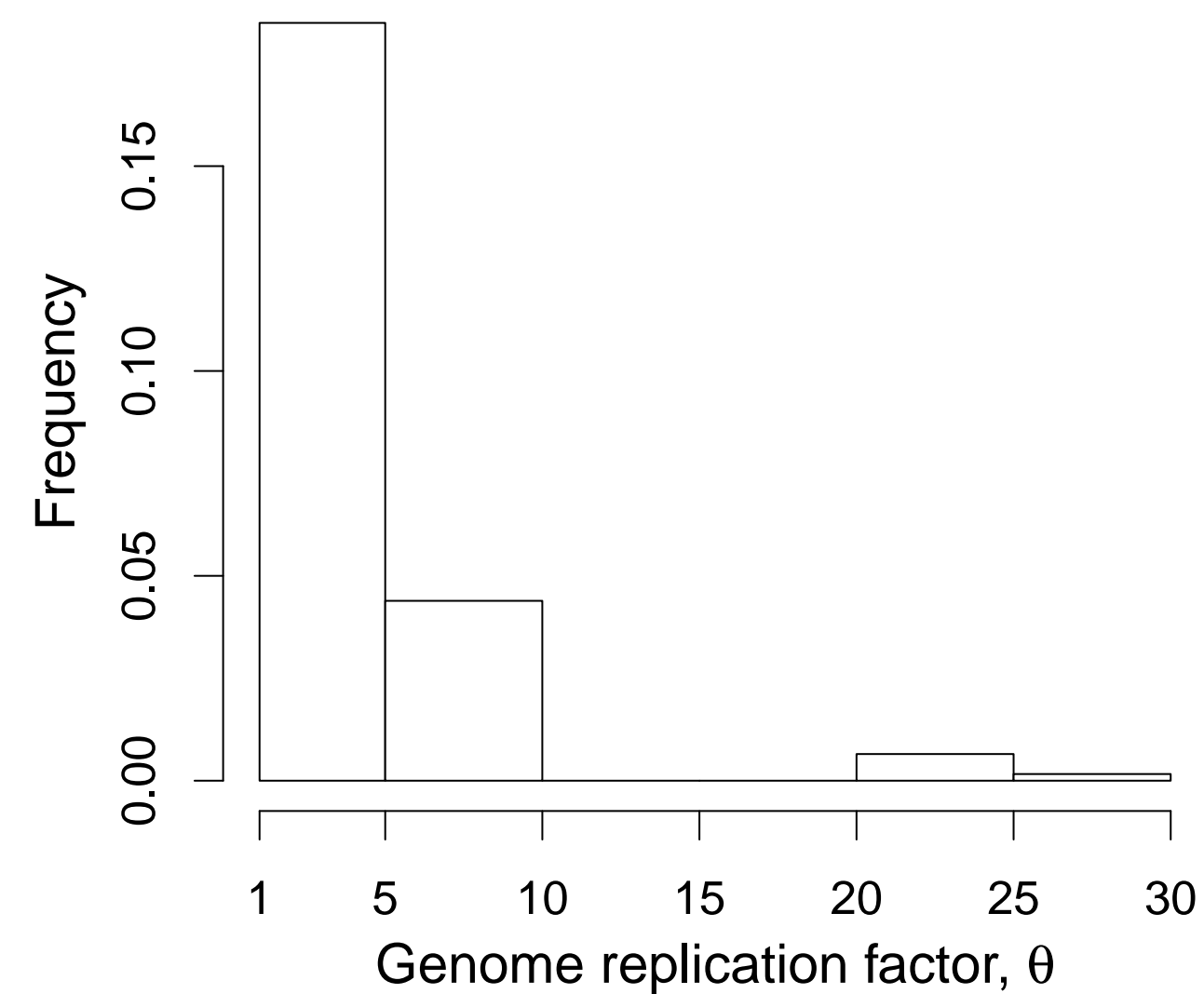

B

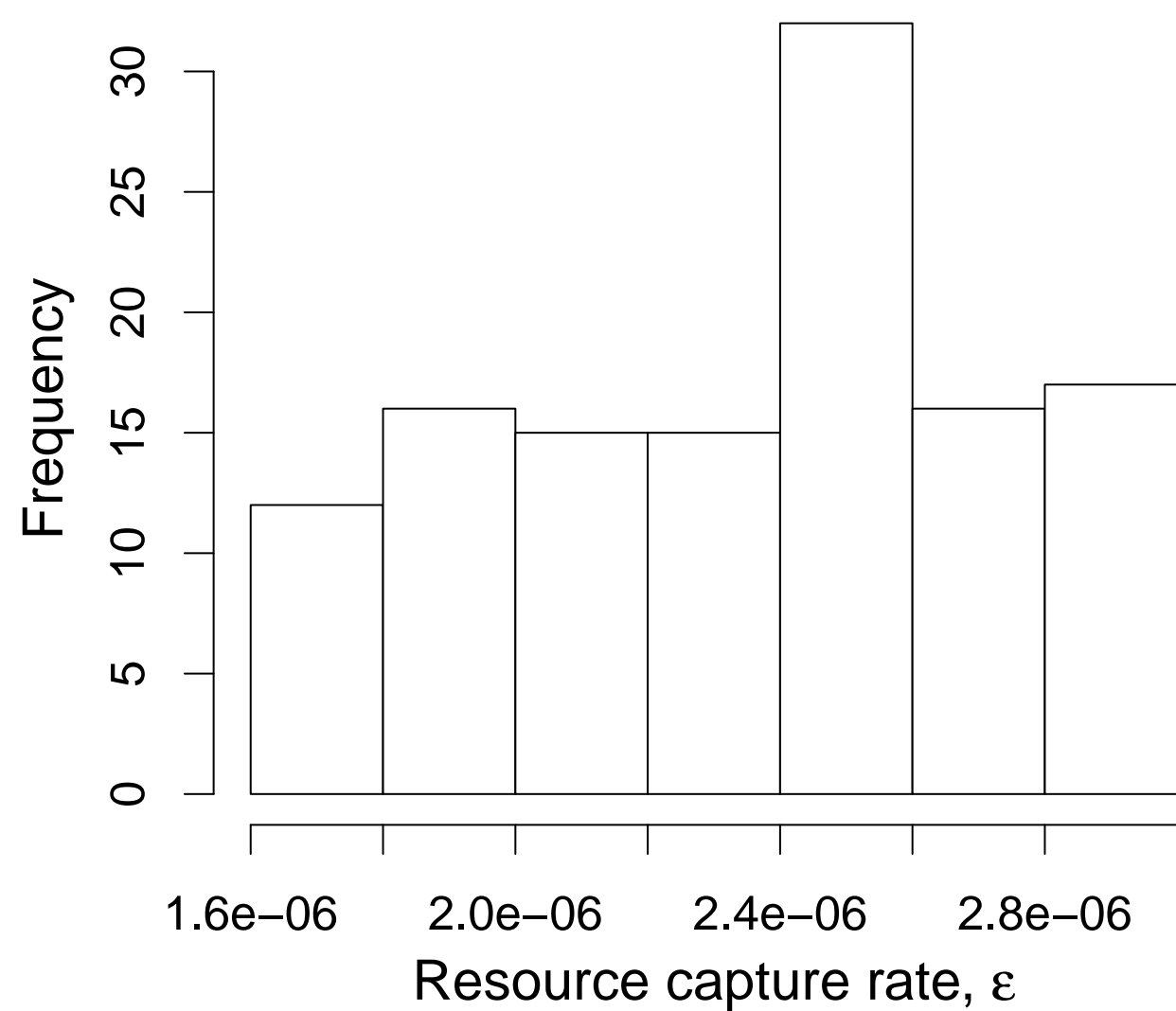

C

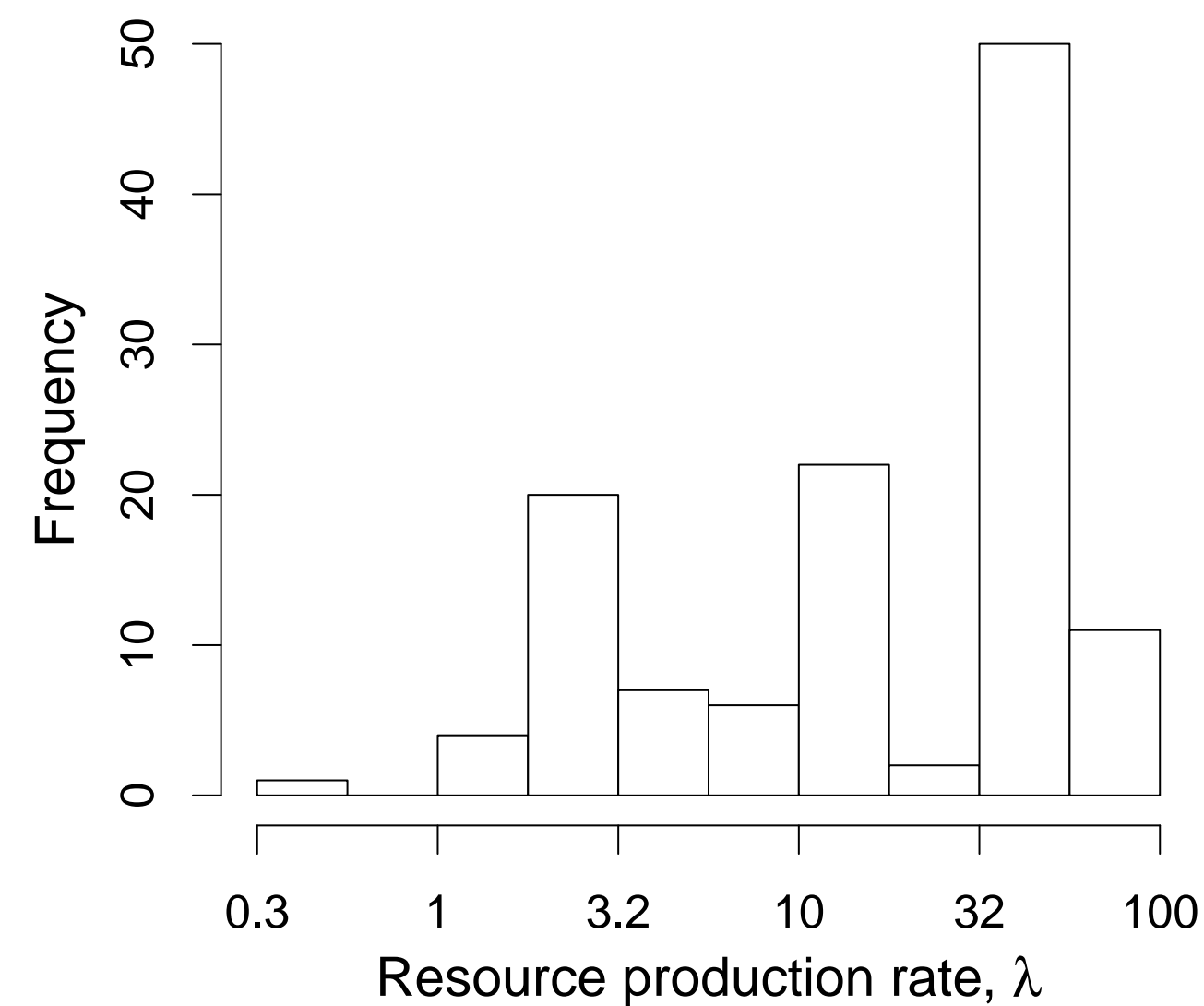

D

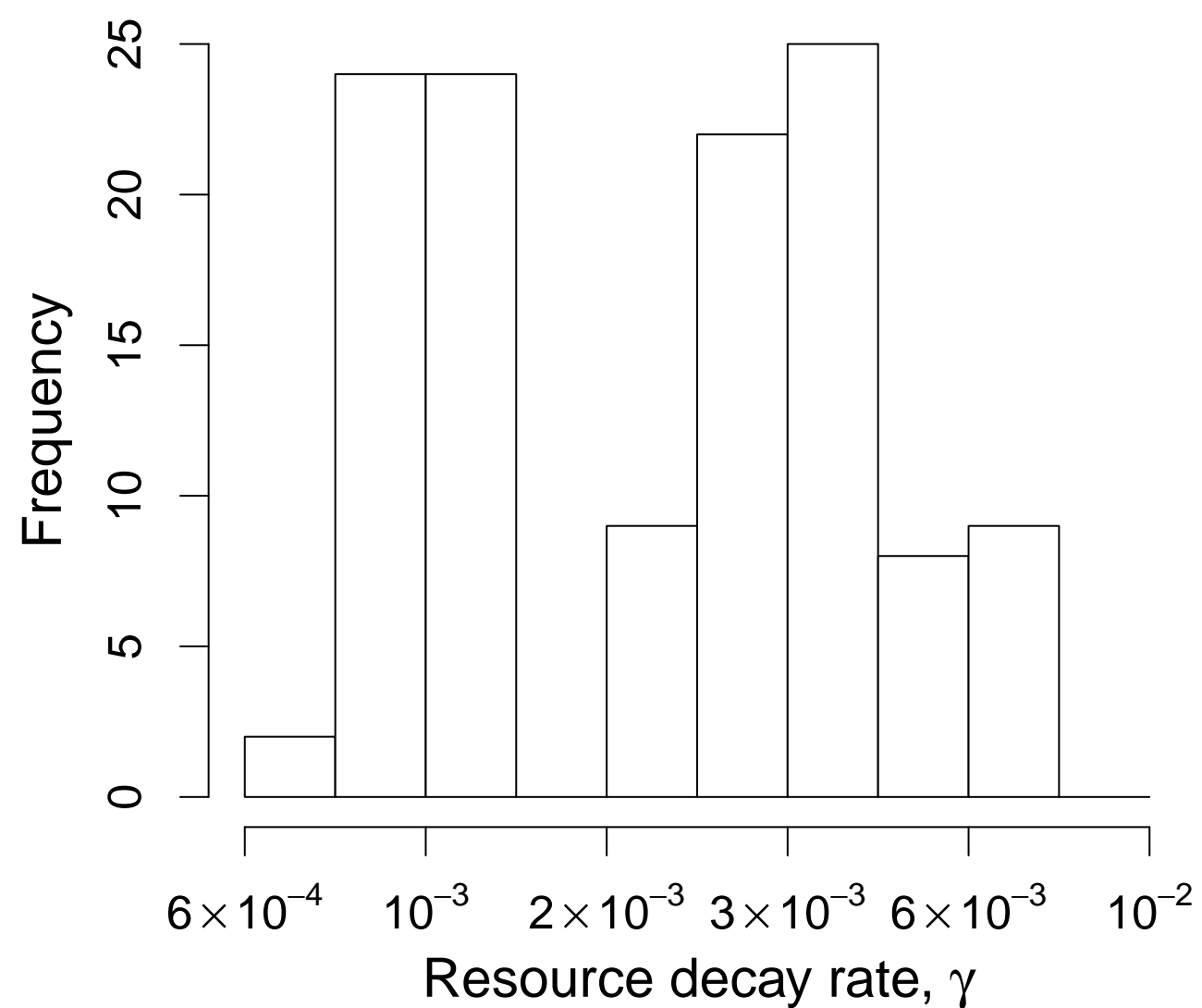

E

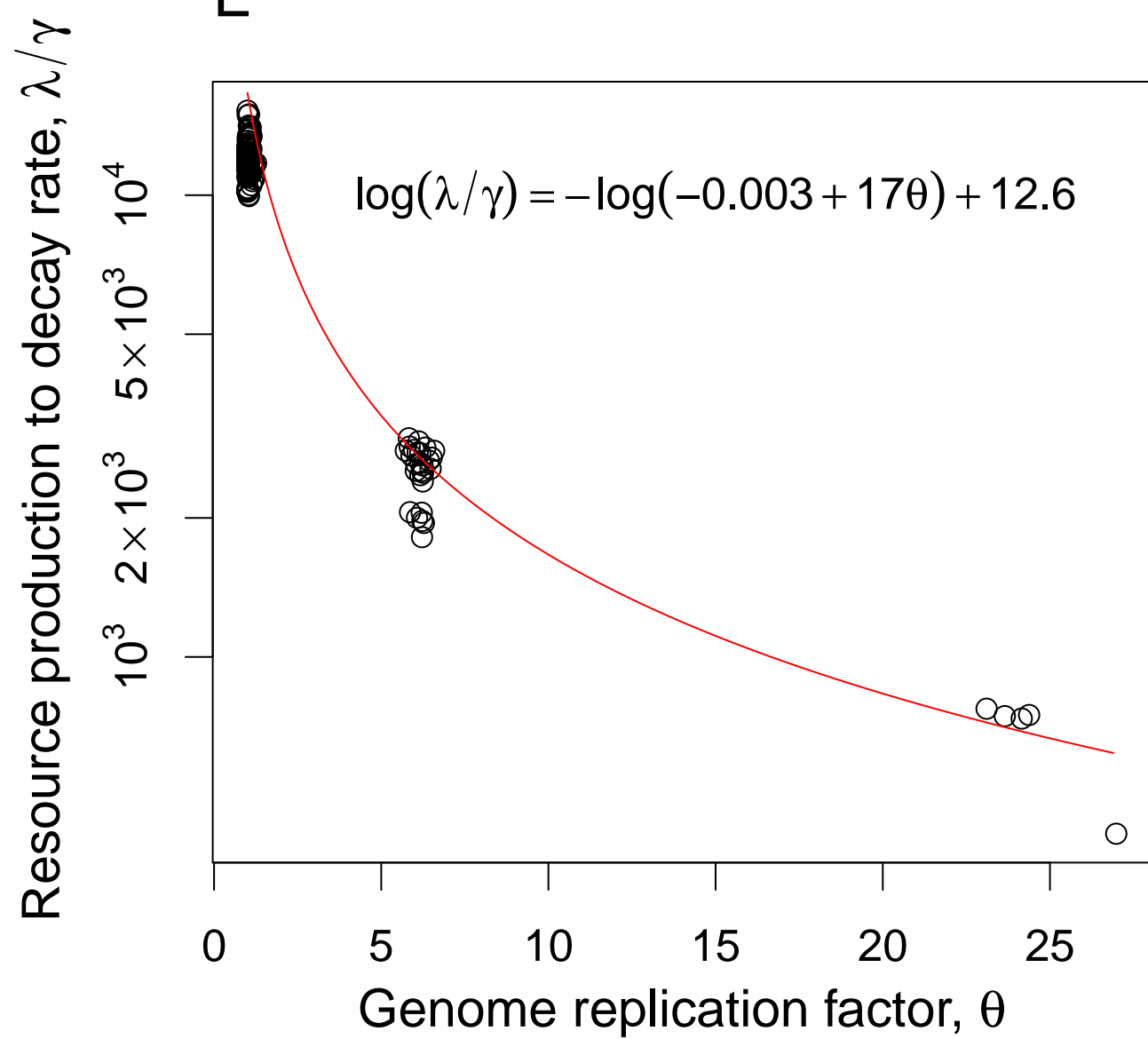

F

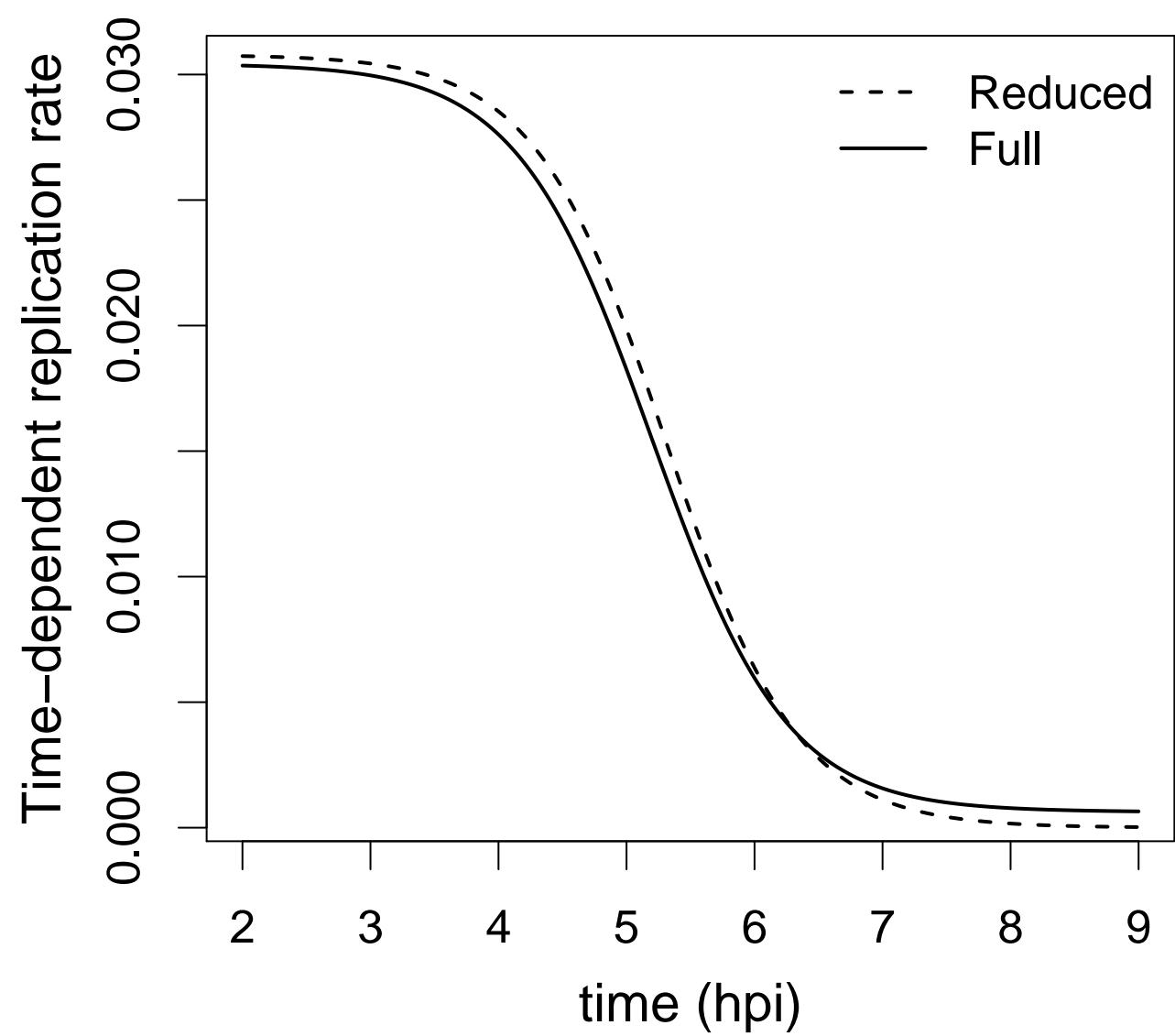

Supplement: S2 Fig — The best 123 estimated values for each parameter are represented. A-D: Histograms of best estimated values for θ (A), ε (B), λ (C) and γ (D). E: Correlation between resource production to decay rate and genome replication factor. The best fit curve is shown in red and its equation is provided (Pearson p-value <2.2 ⋅ 10−16 and R2 = 0.897). F: Time-dependent replication rate given by the reduced model (Λ (t), dashed line) and the full model (θεR(t), plain line). (PDF) [file ppat.1009277.s002.pdf]

Relative WT output

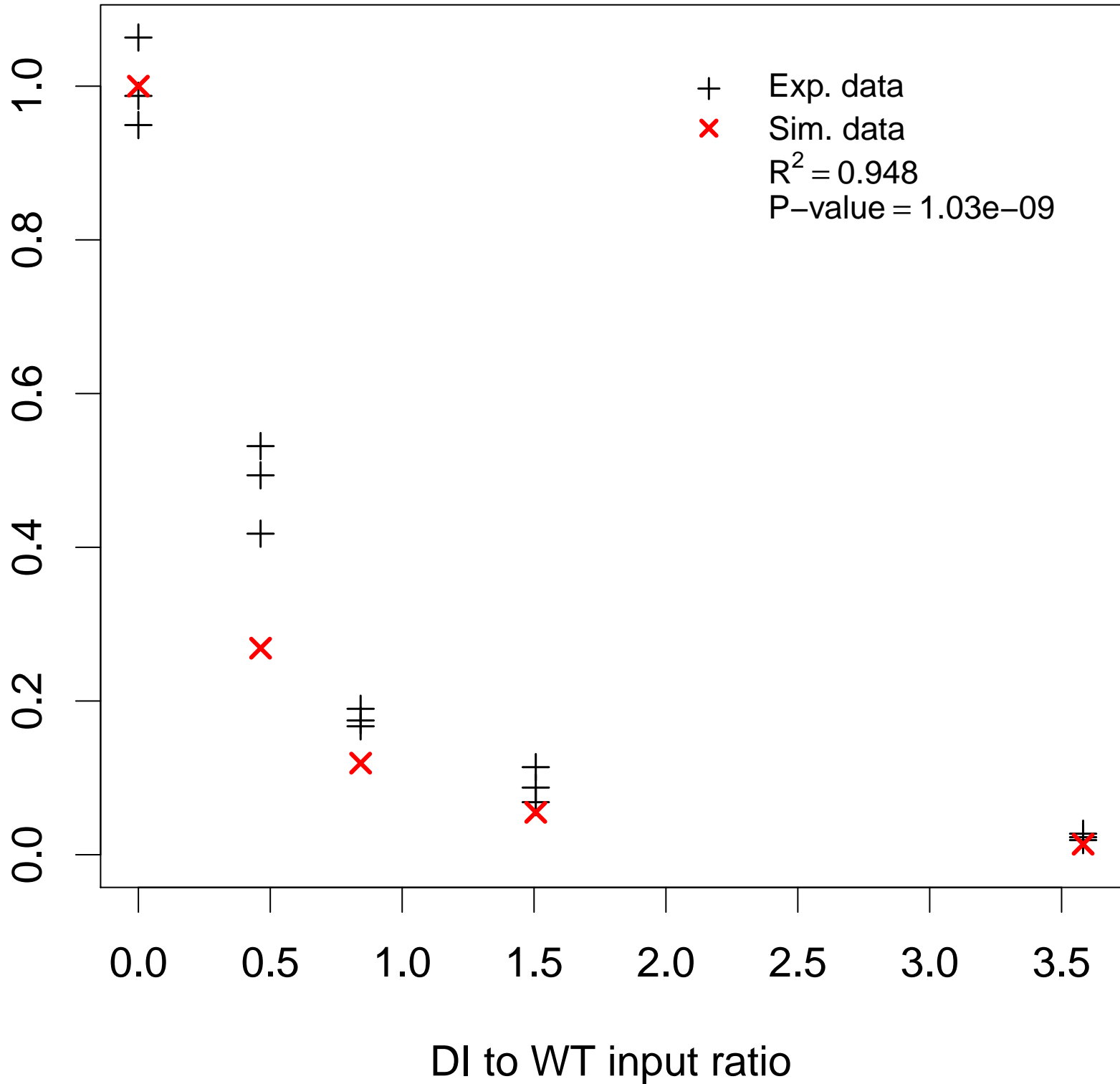

Supplement: S3 Fig — Three experimental replicate values (black dots) of relative WT virus output are represented for various DI to WT input (proxy of multiplicities of infection) ratios. Red dots indicate predicted relative WT output starting with the same experimental input ratios. Experimental WT output corresponds to PFU while simulated WT output corresponds to burst size (number of encapsidated genomes at 9 hours post infection). All outputs were normalized by the output value (or the mean for experimental data) of WT:DI = 1:0 input ratio. R-squared and p-value of a Pearson correlation test between experimental and predicted WT outputs are given in the graphic. (PDF) [file ppat.1009277.s003.pdf]

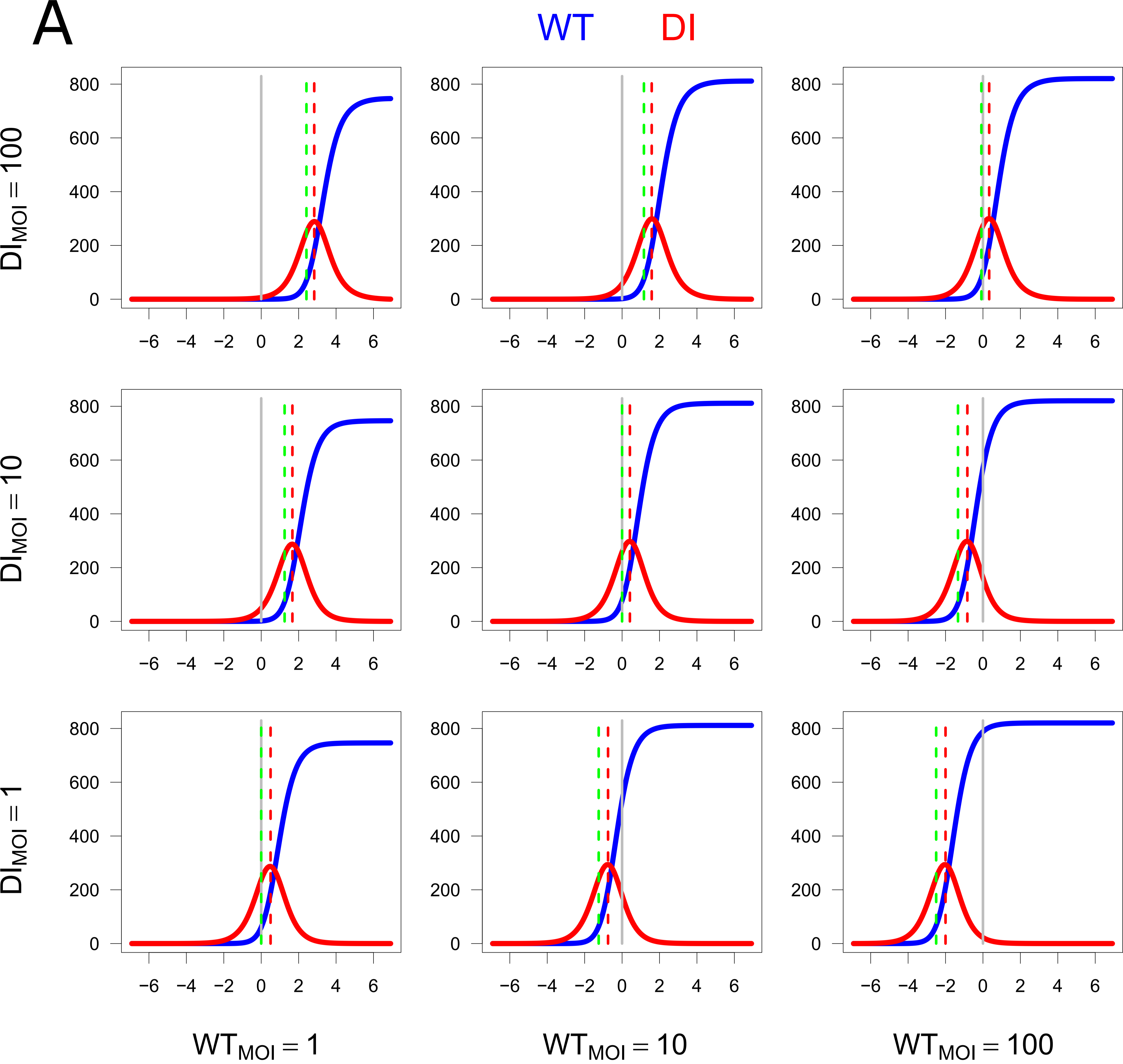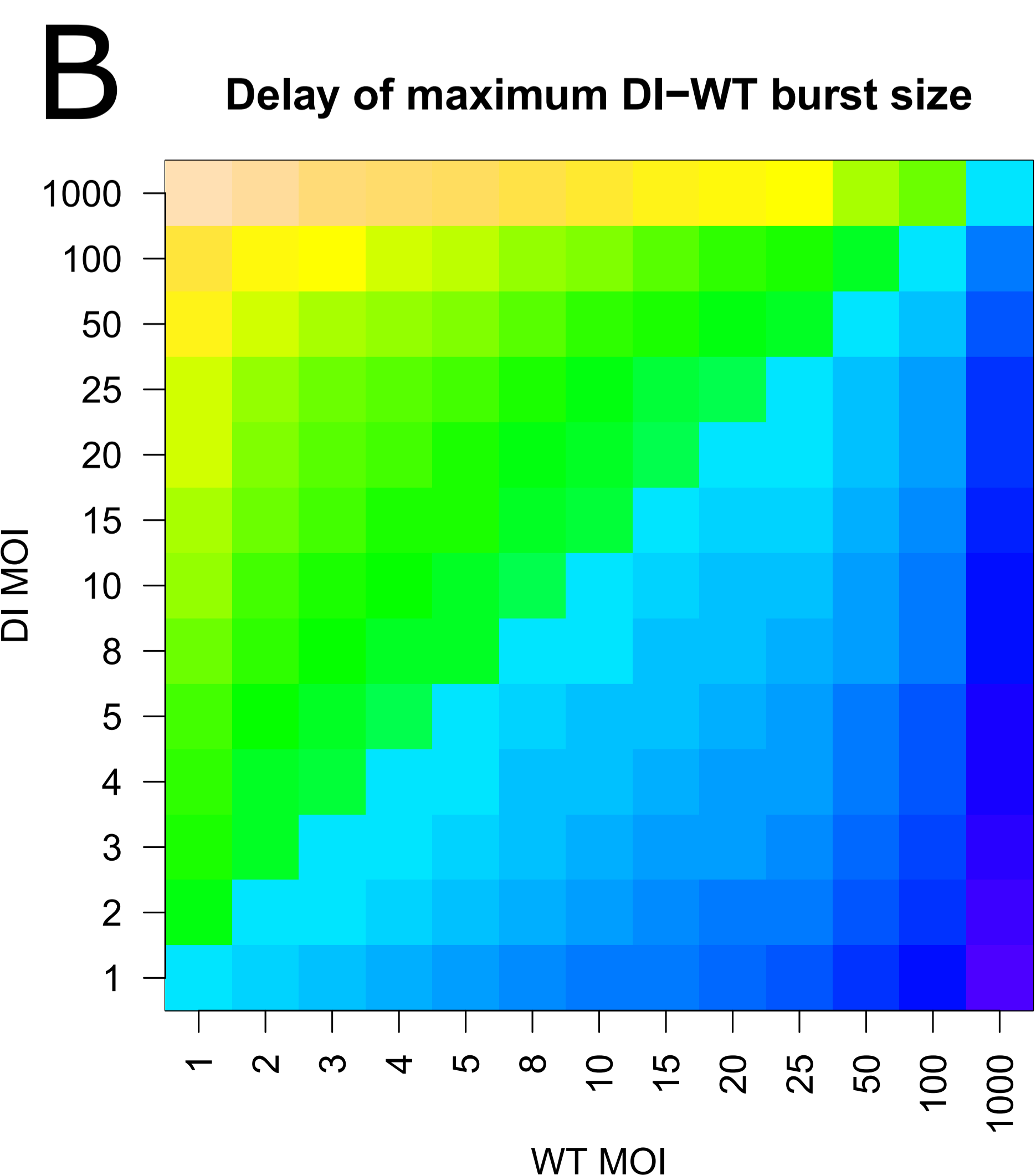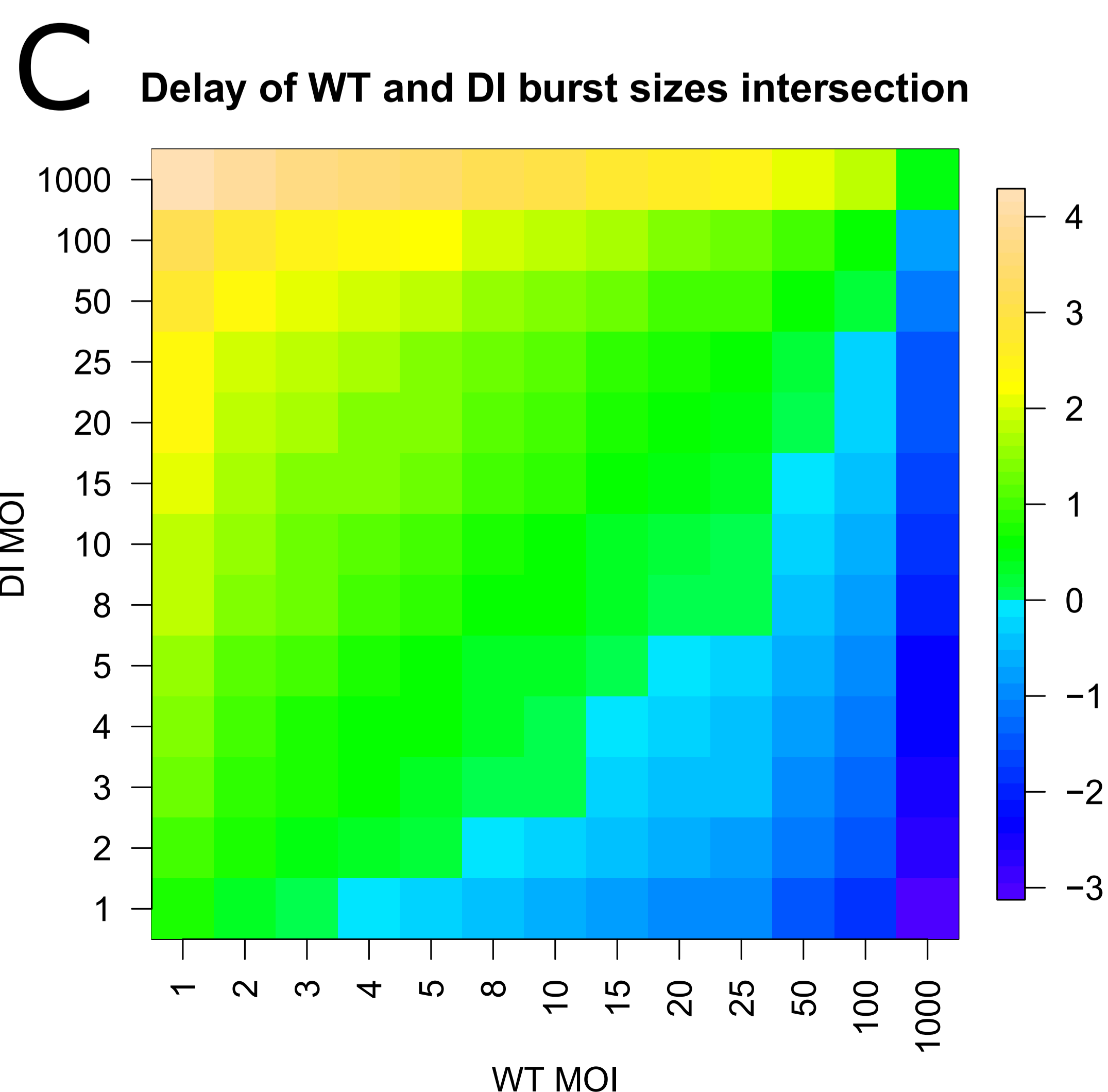

Supplement: S5 Fig — A: Predicted impact of delay for DI particle infection of a cell (x-axis, in hours) on WT (blue) and DI (red) burst sizes (y-axis). One line represents one DI MOI and one column one WT MOI. The grey vertical line indicates no-delay (simultaneous infection). The red vertical line indicates the peak of DI burst size and the green vertical line the maximum difference of DI to WT burst size. B: Heat map of predicted delay for the maximum difference of DI to WT burst size (green lines in A). C: Heat map of predicted delay for WT and DI burst size curves intersection. (PDF) [file ppat.1009277.s005.pdf]

### Dually transfected cell

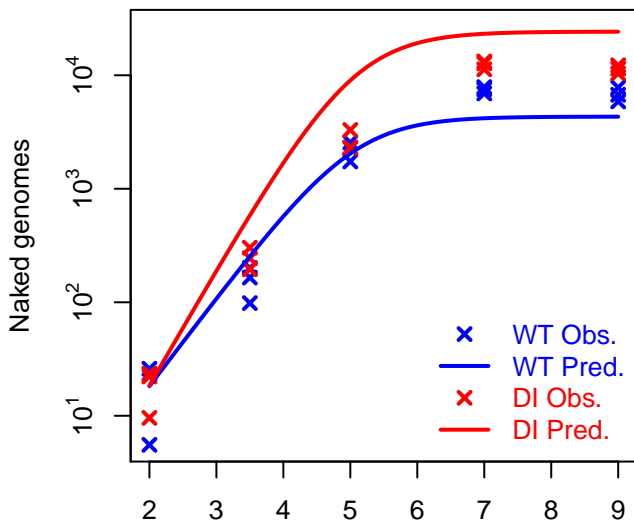

### Singly transfected cell

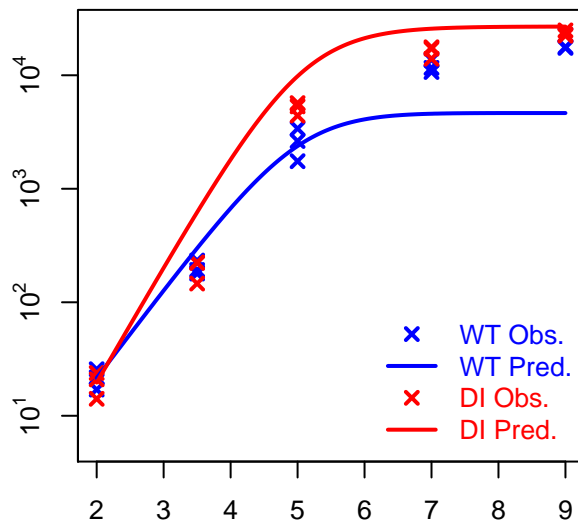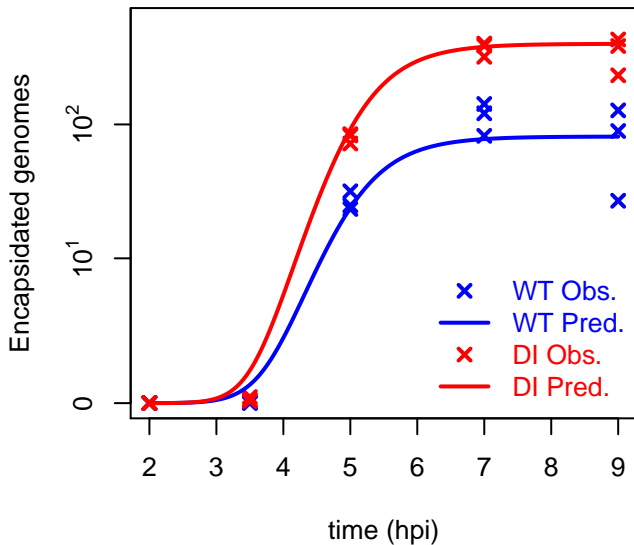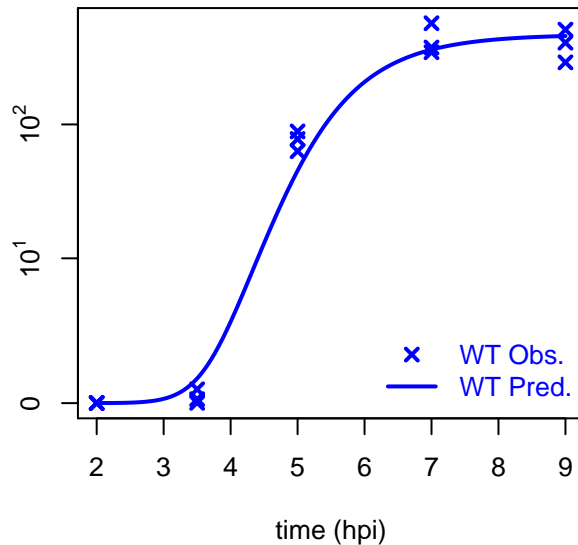

Supplement: S6 Fig — Evolution of the number of WT and DI (A-B) naked genome copies and (C-D) encapsidated genome copies with time, from 2 to 9 hours post transfection (hpt). (A & C) show data in dually transfected cells whereas (B & D) show data in singly transfected cells. WT and DI results are shown in blue and red color, respectively. Crosses indicate experimental data for 3 replicates per sampling time at 2, 3.5, 5, 7 and 9 hpt. Solid curves show the fit of the reduced model with P fixed to the ratio of WT to DI genome lengths (P = 7515bp/5733bp, Eqs (7)–(10)). (PDF) [file ppat.1009277.s006.pdf]
