# Supplementary material for: Experimental and mathematical insights on the interactions between poliovirus and a defective interfering genome
Source: PLoS Pathog. 2021 Sep 27;17(9):e1009277. doi: 10.1371/journal.ppat.1009277 (PMC8496841; doi:10.1371/journal.ppat.1009277)
Supplement: S4 Fig — (PDF) [file ppat.1009277.s004.pdf]

A

"BLIND" OPTIMIZATIONS

Obtain broad boundaries on each parameter *p*:

$$p \in [p_{min}, \, p_{max}]$$

B

OPTIMIZATION OF 9 PARAMETERS ON REDUCED MODEL: P, ω, η, κ, α, β, L, s, t_0

x250

x20

1. Draw random starting values for each parameter *p* in uniform distributions: $p_{start} \sim \mathrm{Unif}(p_{min}, p_{max})$
2. Run Maximum Likelihood optimization based on Least Square score (eq. 11).
   Obtain estimation $\widetilde{p}$ for each parameter.
3. Draw new random starting values for each parameter *p* near previous estimation of step 2:

$$p_{start} \sim \mathrm{Unif}\left(\max(0.95 \cdot \widetilde{p}, p_{min}), \min(1.05 \cdot \widetilde{p}, p_{max})\right)$$

FIX PARAMETERS TO BEST ESTIMATED VALUES

C

OPTIMIZATION OF 4 REMAINING PARAMETERS ON FULL MODEL: θ, ε, λ, γ

Launch steps 1 to 3 of procedure B for the 4 remaining parameters.
