# Supplementary material for: Experimental and mathematical insights on the interactions between poliovirus and a defective interfering genome
Source: PLoS Pathog. 2021 Sep 27;17(9):e1009277. doi: 10.1371/journal.ppat.1009277 (PMC8496841; doi:10.1371/journal.ppat.1009277)
Supplement: S1 Table — For each model, described in S1 Text, the goodness of fit is evaluated by the squared Pearson correlation coefficient between experimental and fitted data (R2), and the quality of the models is evaluated by the log-likelihood (−2 ⋅ log(L)) and Akaike information criterion (AIC) from a linear model between experimental and fitted data. (ZIP) [file ppat.1009277.s007.zip › S1_Table.pdf]

Table S1: **Model selection.**

**Model**	R^2	$\mathbf{-2 \cdot log(L)}$	**AIC**
$\mathcal{M}^{\emptyset}$	0.717	-69.670	-63.670
$\mathcal{M}^{2}$	0.760	-83.567	-77.567
$\mathcal{M}^{23}$	0.760	-83.664	-77.664
$\mathcal{M}^{12L}$	0.970	-257.340	-251.340
$\mathcal{M}^{12}$	0.957	-227.307	-221.307
$\mathcal{M}^{123L}$	0.974	-268.831	-262.831
$\mathcal{M}^{123}$	0.964	-243.349	-237.349

For each model, described in S1 Text, the goodness of fit is evaluated by the squared Pearson correlation coefficient between experimental and fitted data (R^2), and the quality of the models is evaluated by the log-likelihood ($-2 \cdot \log(L)$) and Akaike information criterion (AIC) from a linear model between experimental and fitted data.
