# Supplementary material for: Experimental and mathematical insights on the interactions between poliovirus and a defective interfering genome
Source: PLoS Pathog. 2021 Sep 27;17(9):e1009277. doi: 10.1371/journal.ppat.1009277 (PMC8496841; doi:10.1371/journal.ppat.1009277)
Supplement: S1 Text — (PDF) [file ppat.1009277.s008.pdf]

Supplementary Methods

In this Supplementary Methods, we first describe the model selection procedure. We start by presenting the different models tested and then the statistics used to compare their goodness of fit and quality. In the second section, we present an analytical study of the model, showing that the reduced version of the model is a good approximation of the full version, under given conditions. In the last section, we describe how we estimated the ratio of DI to WT replication rate (P parameter) on the DI mutants experimental data.

Model selection procedure

Description of the models

We built different versions of our model based on the three main features that we hypothesized: (1) the replication and capsid production relies on limiting resources; (2) DI genomes replicate faster than WT genomes; and (3) DI genomes encapsidate faster than WT genomes.

Let us denote the full version of the model, including all three features and presented in the main text (Eqs.(1)–(6)), as $\mathcal{M}^{123}$, and its reduced version, assuming a logistic function for the decrease in resources (Eqs. (7–10)), as $\mathcal{M}^{123L}$. Now let us build a series of models lacking some of these features. We start with the simplest model, lacking all three features and denoted $\mathcal{M}^{\emptyset}$. The equations of $\mathcal{M}^{\emptyset}$ are:

$$\frac{dG_{WT}}{dt} = \zeta G_{WT} - c_g \kappa C G_{WT} - \alpha G_{WT} \tag{S1}$$

$$\frac{dC}{dt} = \eta \zeta G_{WT} - \kappa (G_{WT} + G_{DI}) C - \beta C \tag{S2}$$

$$\frac{dG_{DI}}{dt} = \zeta G_{DI} - c_g \kappa C G_{DI} - \alpha G_{DI} \tag{S3}$$

This model has 5 parameters and is the most basic, with a similar replication rate for WT and DI genomes ζ, a ratio of capsid to genome production by WT η, a similar encapsidation rate for WT and DI genomes κ, and a decay rate of genomes (resp. capsids) α (resp. β).

We now introduce the most obvious feature: the faster replication of DI genomes, based on their shorter size [1]. The model is denoted $\mathcal{M}^2$ and reads as:

$$\frac{dG_{WT}}{dt} = \zeta G_{WT} - c_g \kappa C G_{WT} - \alpha G_{WT} \quad \text{(S4)}$$
$$\frac{dC}{dt} = \eta\zeta G_{WT} - \kappa(G_{WT} + G_{DI})C - \beta C \quad \text{(S5)}$$
$$\frac{dG_{DI}}{dt} = P\zeta G_{DI} - c_g \kappa C G_{DI} - \alpha G_{DI} \quad \text{(S6)}$$

The only additional parameter is P, representing the ratio of DI to WT replication rate, appearing in the equation for DI genomes (G_{DI}). Next, we add either of the two other features to this model. Model $\mathcal{M}^{23}$ assumes both the faster replication (feature 2) and encapsidation (feature 3) of DI genomes:

$$\frac{dG_{WT}}{dt} = \zeta G_{WT} - c_g \kappa C G_{WT} - \alpha G_{WT} \quad \text{(S7)}$$
$$\frac{dC}{dt} = \eta\zeta G_{WT} - \kappa(G_{WT} + \omega G_{DI})C - \beta C \quad \text{(S8)}$$
$$\frac{dG_{DI}}{dt} = P\zeta G_{DI} - c_g \omega \kappa C G_{DI} - \alpha G_{DI} \quad \text{(S9)}$$

It has one additional parameter, ω, representing the ratio of DI to WT encapsidation rate. Finally, model $\mathcal{M}^{12}$ assumes that DI replicates faster than WT (feature 2), and that replication and capsid production rely on limiting resources (feature 1):

$$\frac{dG_{WT}}{dt} = \theta\varepsilon G_{WT} R - c_g \kappa C G_{WT} - \alpha G_{WT} \quad \text{(S10)}$$
$$\frac{dC}{dt} = \eta\theta\varepsilon G_{WT} R - \kappa(G_{WT} + G_{DI})C - \beta C \quad \text{(S11)}$$
$$\frac{dG_{DI}}{dt} = P\theta\varepsilon G_{DI} R - c_g \kappa C G_{DI} - \alpha G_{DI} \quad \text{(S12)}$$
$$\frac{dR}{dt} = \lambda - c_r \varepsilon (G_{WT} + G_{DI})R - \gamma R \qquad R(0) = \lambda/\gamma \quad \text{(S13)}$$

This model has a total of 9 parameters and one new state variable for the resources that are depleted during replication and capsid production. The new parameters are the genome replication factor θ, the resource capture rate by viral genomes ε, the resource production rate λ and the resource decay rate γ. We can link parameter ζ of the previous models relaxing the assumption of feature 1 (the presence of limiting resources required for replication and capsid production) to the parameters of this last model $\mathcal{M}^{12}$ including this feature, by noting that at time t=0: $\zeta = \theta\varepsilon R(0) = \theta\varepsilon\lambda/\gamma$. As for the full model including

the three features $\mathcal{M}^{123}$ presented in the main text, we can build a lower dimension version of model $\mathcal{M}^{12}$ by assuming that the decrease in resources due to viral uptake for replication and capsid production follows a logistic decreasing function. The reduced model, denoted $\mathcal{M}^{12L}$, reads as:

$$\frac{dG_{WT}}{dt} = \Lambda(t)G_{WT} - c_g\kappa C G_{WT} - \alpha G_{WT} \tag{S14}$$

$$\frac{dC}{dt} = \eta\Lambda(t)G_{WT} - \kappa(G_{WT} + G_{DI})C - \beta C \tag{S15}$$

$$\frac{dG_{DI}}{dt} = P\Lambda(t)G_{DI} - c_g\kappa C G_{DI} - \alpha G_{DI} \tag{S16}$$

$$\Lambda(t) = \frac{L}{1 + e^{s(t-t_0)}} \qquad L \approx R(0) \tag{S17}$$

Optimization and comparison of the models

The optimization of each model was conducted iteratively, as described in the main text. Models $\mathcal{M}^{\emptyset}$, $\mathcal{M}^{2}$ and $\mathcal{M}^{23}$ were optimized in one step (20 times 250 optimizations), whereas model $\mathcal{M}^{12}$ was optimized in two steps, first estimating the parameters of reduced model $\mathcal{M}^{12L}$, and then fixing the common parameters between the two models (κ, α, β, P) to estimate the remaining parameters of model $\mathcal{M}^{12}$ in a second step (θ, ε, λ, γ).
For each model, the 150 best optimized sets of parameters were retained and used to compute the squared Pearson correlation coefficient between experimental and fitted data (R^2), and the log-likelihood ($-2 \cdot \log(L)$) and Akaike information criterion (AIC) of a linear model between experimental and fitted data. The values of these statistics are presented in Table S1. Although two versions of the model ($\mathcal{M}^{12L}$ and our reduced model $\mathcal{M}^{123L}$) were predicted to perform slightly better than our full model ($\mathcal{M}^{123}$, Eqs.(1)–(6)) based on tested statistics, we decided to keep our full model because it visually fits better the experimental data, reproducing its most important characteristics. Additionally, its statistics are very good.

Analytical study of the model

Let us start with the full version of the model. At early times of infection of a cell, because resources are abundant and because the formation of capsids requires genome templates, we assume that the terms for encapsidation are negligible compared to genomic replication.

We also assume that genome decay is negligible. From eqs.(1) and (6), we thus assume that:

$$\begin{aligned} \alpha + c_g \kappa C(t) &<< \theta \varepsilon R(0) \\ \alpha + \omega c_g \kappa C(t) &<< P\theta \varepsilon R(0) \end{aligned}$$

with $R(t) \equiv R(0) = \lambda/\gamma$. From eqs. (1) and (3), we get:

$$G_{WT}(t) = G_{WT}(0) e^{\theta \varepsilon R(0) t} \quad \text{(S18)}$$

$$G_{DI}(t) = G_{DI}(0) e^{P\theta \varepsilon R(0) t} \quad \text{(S19)}$$

At $P > 1$, soon $G_{DI} >> G_{WT}$, hence:

$$G_{DI} + G_{WT} \approx G(0) e^{st} \quad \text{(S20)}$$

with $s = P\theta\varepsilon R(0) = \dfrac{P\theta\varepsilon\lambda}{\gamma}$. With eq. S20, eq. (4) takes the form:

$$\frac{dR}{dt} = \lambda - (Ae^{st} + \gamma)R \quad \text{(S21)}$$

with $A \equiv c_r \varepsilon G(0)$. The solution of eq. S21 using the standard method is:

$$R(t) = R(0) \left[1 + \gamma \int_0^t e^{\frac{A}{s} e^{st} + \gamma t} \right] e^{-\frac{A}{s} e^{st} - \gamma t} \quad \text{(S22)}$$

At $t = 0$, $R(0) \approx \lambda/\gamma$. At $t >> \dfrac{1}{s} \log \left(\dfrac{\gamma}{A} \right)$, we get:

$$R(t) \approx R(0) \frac{\gamma}{\gamma + Ae^{st}} = \frac{R(0)}{1 + e^{s(t-t_0)}} \quad \text{(S23)}$$

with $t_0 \equiv \dfrac{1}{s} \log \left(\dfrac{\gamma}{A} \right) = \dfrac{\gamma}{P\theta\varepsilon\lambda} \log \left(\dfrac{\gamma}{c_r \varepsilon G(0)} \right)$.

For very long times, depletion of resources at $t > t_0$ ($t_0 = 181$ minutes with parameter values from Tab 1) causes the modification of eq. S20, because $R(t) << R(0)$, and eq. S23 ceases to be valid. We arrive therefore at a low steady state.

Hence, the approximation of the full model with its reduced version is valid on a short timescale only. The use of the reduced model is justified by the fact that we simulate it over 9 hours only, the time of a replication cycle before burst of the cell.

References

1. Rouzine IM, Weinberger LS. Design requirements for interfering particles to maintain coadaptive stability with HIV-1. Journal of virology. 2013;87(4):2081–93. doi:10.1128/JVI.02741-12.
