# Supplementary material for: Experimental and mathematical insights on the interactions between poliovirus and a defective interfering genome
Source: PLoS Pathog. 2021 Sep 27;17(9):e1009277. doi: 10.1371/journal.ppat.1009277 (PMC8496841; doi:10.1371/journal.ppat.1009277)
Supplement: Attachment — Submitted filename: Poin-by point responses PLoS Pathogens DIP.docx [file ppat.1009277.s009.docx]

Reviewer's Responses to Questions

**Part I - Summary**

Please use this section to discuss strengths/weaknesses of study, novelty/significance, general execution and scholarship.

Reviewer #1: There is a renewed interest in defective interfering (DI) particles (and other types of defective genomes) because they are produced and maintained in populations of many viruses (particularly RNA viruses), and because they may play regulatory roles, particularly in the interaction with the host immune system. In the present study, experimental data on WT and DI poliovirus are presented, and confronted with a relatively simple model (a reduced model to determine some parameters and a full one) of differential equations, based on one previously developed for HIV-1. The model considers as central parameters rates of replication and encapsidation to explain the outcome of WT-DI competitions. The predictive value of the model is tested with some experimental results, and reasons for agreements and disagreements are discussed.

Although the study is of potential interest, in its present form it fails to relate the findings to previous work on DI particles, and models on interference mechanisms. The authors fail also to underline the major conclusions, novelty, and implications of the experimental results and model. In addition, some experimental protocols should be described in greater detail.

1. Other previously described models and their predictions should be compared with the differential equation-based model presented in the study (Frank, J. Theor. Biol. 2000; Stauffer Thompson et al. J. Gen. Virol. 2009; Laske et al. Virus Res. 2016; Kirkwood and Bangham, PNAS, 1994; among others). For example, the deterministic model of Kirkwood and Bangham emphasizes unpredictable effects of DI activity, a point not discussed in the present study. The merits of the new model relative to previous ones, and its advantages for future work should be explained.

We now extensively discuss previous modelling efforts in the introduction (lines 84-95 of the new version).

2. According to Figure 5 legend the poliovirus used was type 3 (PV3), while according to the Supplementary Methods the WT was PV3 while the DI was constructed on PV1. The poliovirus used as WT and for DI construction should be explained and, if different viral types were used, a justification is needed.

We have added the requested information on the PV type used in the experiment in the legend of Fig. 5. We have also added a sentence to explain the reason why we used WT PV3 and PV1-DI for the co-passaging experiment at lines 315-317.

Also, how the present DI structure compares with others previously characterized for poliovirus should be indicated.

We now describe in lines 66 and subsequent, that poliovirus DIs are in-frame deletion in the capsid-encoding region, but the deleted regions can be variable and different among individual DIs. We have modified the sentence to clarify the point (line 66).

3. A critical experimental value is the proportion of encapsidated RNA measured by resistance to a RNase mixture. Controls (or literature references of previous work by the authors where the controls are described) consisting of mixtures of encapsidated (protected) purified particles and free viral RNA (unprotected) to evaluate the reliability of the quantifications should be described. Indicate also the proportion of cells that were transfected.

RNase-resistant DI genomes were detected only in the double-infected sample with capsid-encoding WT genomes, but not in the DI single-infected sample, although both samples contain detectable DI genomes before RNase treatment (Fig. 1CD). These results indicate that under the experimental conditions used only encapsidated genomes are detected following RNase treatment, but not naked genomes. We have included this information at lines 149-154. The proportion of cells that were transfected has also been included at line 140.

4. There is some confusion about what the authors mean by “replication rate”. Having a shorter RNA, DIs are expected to complete a round of template copying earlier than WT RNA. This does not imply that the replicase (or replicative complex or organelle) travels faster along the RNA template when copying DI RNA than WT RNA. Unless a different rate of polymerase progression has been documented, it would be better to refer to replication being completed in a shorter time for DI than WT. An additional ambiguity becomes apparent when the authors state that “replication rate” in their model includes also translation of the positive sense RNA genome. To complicate matters, in section 4.1 the authors suggest that the DIs will replicate faster than the WT by a factor given by the ratio of WT to DI genome lengths, but the observed discrepancy may be attributed to the various processes linked to replication (which processes in addition to translation mentioned earlier?). In this section they also refer to “replication speed”.

We have reworded the paragraph in the Results section (lines 194-198) that relates to replication, and hope it is more clear now. The replication rate is defined as the product 𝜃𝜀R(t) (units min^-1^). Indeed our assumption is that DIs complete a round of template copying earlier than WT because of their shorter genome length, and not that the polymerase travels faster along DI RNA. We have also gone through the whole text and made some slight rewording when necessary.

We have removed the mention of translation of the positive sense RNA genome in the Results section, which complicated the model description and was not essential. We still refer to it in the Discussion, to explain the discrepancy between P and the ratio of DI to WT genome length.

We have changed the term ‘replication speed’ for ‘replication rate’.

5. The data presented in Fig. 5 require additional information. In panel A, the passages produced a number of mutant DIs. The reader assumes that no mutations occurred in WT but this should be stated. Fitness of variant DIs is mentioned but no information on their values and how was fitness defined and determined is provided. DI mutant 1952 in panel D is named 19522 in panel C. The meaning of the volumes given in panel B PV3 should de explained. The differences between panels E and F and their significance are not clear.

The use of the term ‘fitness’ was removed as what we actually estimate is the DI-to-WT replication ratio P. We made changes to the text accordingly.
The question on mutations in WT PV3 is very interesting. Indeed, we did observe mutations in PV3 occurring during the passages. These mutations likely reflect the process of co-adaptation during WT and DI co-infection. Given that the goal of this experiment was to identify mutations that increase DI replication fitness, we have decided not to include this data in the current manuscript. We plan to present a more complete report on this issue in a future publication focusing on co-adaptation. We have removed panel E to simplify the figure.

6. While the Results and figure legends will benefit from expansion, the Discussion is very long and with speculative arguments. At times, it is considerably naïve, for example when referring to evidence of dengue coinfections in India, when the literature includes many examples of coinfections. It is unlikely that the non-specialist reader can capture the main conclusions and novelty of the present study among a quite disorganized amount of information, mainly in the Discussion.

We have extensively revised the text, figure legends and discussion to address all reviewer’s concerns.

7. At times the distinction between what is predicted by the model without experimental verification and what has been experimentally studied is not clear. While the data in Figure S2 are an experimental verification of model predictions, those in Figures 6 and S4 do not seem to have an experimental verification with the poliovirus system. If this is correct, it should be indicated.

Figure 6 and S4 (now S5) are indeed theoretical results from our model. We made some changes in the legends of these figures to indicate it.

Reviewer #2: Summary:

The presented manuscript `Experimental and mathematical insights on the interactions between poliovirus and a defective interfering genome’ aims to investigate the intracellular time course dynamics of poliovirus and its defective viral genomes (DVGs) that interfere with the full-length virus (FLV) via two mechanisms. First, DVGs are replicons which need FLV’s capsid to form particles, thus hampering encapsidation of the FLV. Second, DVGs replicate more rapidly, thus depleting cellular resources at the expanse of the FLV. Briefly, the authors used a mathematical model to fit the time course genome concertation data of FLV and DVGs to estimate the model parameters. They conclude that the most important parameters that determine the outcome of FLV and DVG competition are DVG-to-FLV replication ratio, P, and DVG-to-FLV encapsidation ratio, omega. The authors then use additional experimental data to validate their model predictions.

Strengths/Weaknesses:

I personally find the idea of combining the experimental data on FLV and DVGs with mathematical modelling interesting and sufficiently novel. The study addresses a rather unexplored problem of numerical quantification of DVG parameters during the FLV-DVG competition as these could help to better design treatment protocols should DVG research progress to a phase of therapy development in the future, particularly the optimal dose and the time of DVG administration. On the other hand, successful DVG infection would have to be secured. Cell transfection is a limitation and it is unclear to me whether FLV-DVG multiple-cycle infection dynamics would be possible to track by the authors. The authors also present their assumption about the model based on the knowledge of their DVGs as a result claiming that the model predicts P and omega to be greater than 1 (e.g. Discussion, lines 386-387). If P=omega=1, there’s no competitive advantage and the outcome will depend on the initial conditions/time of delivery. Computational methods need to be described in more detail.

Reviewer #3: PPATHOGENS-D-21-00004_reviewer

Summary

(Given how covid has changed my working life, I'm reviewing this as I would review a preprint I need. As in, does it make sense, is it basically sound, and do I understand what they're saying? If yes to all three, then I don't think it needs much revision. So I read carefully, but I'm not looking to nitpick in this review.)

This is a really nice example of model and experiment working together. The question of interfering particles is important to understanding viral fitness, the experimental methods are clean, and the modeling is insightful and simple but not-too simple and directly relevant to the experiments. And the model makes successful, non-trivial out-of-sample predictions. Any modeling paper that actually predicts something it didn't fit to is good work (and much more rare than it should be!)

I'm familiar with the both the virology and model, but much better equipped to critique model methods than lab. The model is reasonable, as is the optimization procedure to fit it, and the results are clearly communicated.

In general, the paper is clearly written. I felt like I knew exactly what the paper was about from the intro and why you were interested in it on the first read (with one minor caveat below). This impression held up through the rest of it.

**Part II – Major Issues: Key Experiments Required for Acceptance**

Please use this section to detail the key new experiments or modifications of existing experiments that should be absolutely required to validate study conclusions.

Generally, there should be no more than 3 such required experiments or major modifications for a "Major Revision" recommendation. If more than 3 experiments are necessary to validate the study conclusions, then you are encouraged to recommend "Reject".

Reviewer #1: See Part I

Reviewer #2: The presented mathematical model is a modification of a model from Rouzine and Weinberger (2013) in which a new variable was introduced to simulate cellular resource depletion by FLV and DVGs. I have the following questions regarding modelling, and I list them point by point:

1) Have the authors tried to fit the model with a fixed P? There is a note in the manuscript that the estimated ratio could correspond to 1.311 based on the FLV and DVG genome lengths. This would lower the dimensionality of the fitting problem. How would the predictions change?

We ran an optimization of the reduced model with a fixed P (P=7515/5733), and this is what we obtained (see figure below).

As P is fixed to a larger value than the one optimized in our article (1.075), we can see that the model does not reproduce well the evolution of naked genomes in both dually and singly-transfected cells. The large value of P forces a predefined gap between the number of WT and DI naked genomes, resulting in overestimation of DI naked genomes and underestimation of WT naked genomes.

We also tried to optimize the full model with a fixed P, but the optimization did not work well, the estimated parameters yielded aberrant simulations. It would still require our two-step approach (reduced model first, then full model), hence it does not help with our dimensionality issue. This is because the unknown variable is R, the cell resources, and fixing P does not directly inform on variable R, hence it does not help with the full model optimization.

We added the figure above to the manuscript (Fig S6), as well as one sentence in the discussion with this additional information, lines 443-444.

2) What is the reason to assume that DVGs encapsidate faster than FLV? How do the predictions change if no such competitive advantage is assumed for DVGs?

Our experimental results indicate that DVG replicates faster than WT. If DVG and WT are replicating in the same replication complex, it is possible that DVG will be more effective at consuming capsid proteins at the assembly line. In addition, the shorter genome of DVGs could facilitate encapsidation, however, this remains to be determined.

In Supplementary Methods, we present model 𝓜^12^ that assumes the same encapsidation rate for both WT and DI genomes (i.e. omega=1). We inserted a new Supplementary figure, Fig S1, that shows model 𝓜^12^ fit to the experimental data. With this assumption, we can see that the model underestimates the number of encapsidated DI genomes, as expected. We added one sentence in the manuscript (section 3.3) to describe these results.

Because we added a new FigS1, all subsequent supplementary figures were renamed accordingly.

3) When fitting the model, both naked and encapsidated genome abundancies define the objective function to be minimized by least squares. I am not an experimentalist and don’t understand the protocol much, but how do the authors differentiate between encapsidated genomes and empty capsids (e.g. Figure 3)? Are the data in Fig. 3 truly just encapsidated genomes? I think this is important to also write in the text as modellers could struggle to find this information in there.

We counted the numbers of RNase-resistant (encapsidated) ‘genomes’ (genomes included in capsids) by ddPCR, so the numbers did not include those of ‘empty’ capsids. We have clarified this point at lines 149-154.

4) What is the authors’ opinion on the possible assumption that DVG uses less cellular resources per genome than FLV, possibly due to its smaller size? Is that something they would consider in their model?

We had to make the most parsimonious assumption, because we do not measure resource dynamics. Identification of "cellular resources" would be necessary to test this assumption experimentally, but this is difficult to address. In principle, the DVG shorter genome should be approximately ⅓ more “economical” with respect to consumption of nucleotides, but it is likely that all other resources needed for virus replication would remain fairly similar. However, given that both genomes replicated in the same cells and are employing the same resources both of them should be limited by limited resources in a similar way. Our data and our model indicate that both DI and WT indeed replicated at a lower level, and given that WT replication is reduced, at expense of the DI replication, then the co-infection attenuates infection by WT. This is reflected on the model and on the experimental data.

5) Line 222: Predictions also demonstrate that DVGs are more efficiently encapsidated than FLV (omega=2.185). I disagree with this sentence because omega>1 is the assumption the authors make, not a prediction. Can the model reproduce the data when omega=1? See my comment above.

P>1 and omega>1 are experimental observations and not assumptions, perhaps due to the shorter DI genome length. The estimated values of P and omega result from the model fitting, and they are indeed >1.

We did some modifications to the text to avoid any confusion in this regard.

“DI genomes are assumed to encapsidate faster” was changed to “DI genomes are set to encapsidate faster [...], based on experimental observations from Fig 1C&D” (lines 194-195).

“Predictions also demonstrate” was changed to “Model predictions are also consistent with the experimental observation ” (line 243-244).

We have answered the question about the case where omega=1 above, see question 2.

6) Line 226-227: Again, not a prediction, interference is an assumption in the model which allows to fit the data.

Similarly to what is stated in the previous answer to question 5, interference is an experimental fact that was built into the model, it is not an assumption. We modified the sentence to make it more clear: “Thus, it predicts a strong interference between DI and WT” was changed to “Thus, it reproduces the strong interference experimentally observed between DI and WT”.

7) In the equation (10) for Lambda(t), the factor s should have units of min^-1 and not be unitless as (t-t0) is in mins and L is unitless. Or is it L that should have units?

This is correct, s should be in min^-1^, and L should be in min^-1^ as well. We have updated the units in Table 1. We have also changed the sign of s in Table 1 and removed the minus in equation 10 in order to simplify things.

8) To be honest, I don’t follow the model reduction procedure. When I look at the models (1)-(4) and (7)-(10), they should at least yield the same units, that is genomes/time or capsid/time. When I look at eq. (2) for capsid dynamics, then e.g. the rate (eta x Lambda(t) x G_wt) has units (capsid x genome^-1 x nothing x genome) which results in just capsid as units, given Table 1. For this reason, the rates are not comparable. Also, lines 236-237, these time-dependent rates 3.07x10^-2 and 3.02x10^-2 are unitless?

We have corrected the unit of L, as mentioned in the previous point, hence now Lambda is in the right unit (min^-1^) and the units of the reduced model should also work.

The time-dependent rates in lines 236-237 (264 of the new version of the manuscript) are in min^-1^, we have added the units in the text, thank you for reporting this.

9) Figure S2: Is this a reasonable validation? This is related to my question about free capsids – if the quantification of encapsidated genomes accounts for free capsids, PFUs might not correspond and normalization will mask this.

As described above (3), the quantification of encapsidated genomes did not account for free capsids.

10) How exactly are the data from Figure 1 used in modelling? This part is a bit unclear to me.

The data in Fig 1C&D are used to estimate model parameters. We have also added the information in the legend of Fig 3 (“data drawn from Fig 1C&D, see Material and Methods for details”). The information is also present in the text in the Results section: “Model predictions fit well the experimental measurements in Fig 1 C&D”. We also modified the first sentence of section 5.3 in Material and Methods: “The model was fitted to the experimental data of Fig 1C&D in order to estimate model parameters describing our biological system.”.

We explain how these data are used in the modelling in section 5.3 of Material and Methods (lines 682-703) we have added reference to Fig1 to make it more clear): “Raw experimental data are WT and DI total RNA copy number (g^tot_WT and g^tot_DI, Fig 1C) and RNAse treated genome copy number (v_WT and v_DI, Fig 1D). The former corresponds to the total number of genomes (naked and encapsidated) and the latter to the number of encapsidated genomes. The numbers of WT and DI naked (i.e.non-encapsidated) genomes are thus: g_WT=g^tot_WT−v_WT and g_DI=g^tot_DI−v_DI. Additionally,...” (see the end of the corresponding paragraph for all the information)

11) Figure 5: What exactly is this figure saying? If I understand correctly, 8 DVGs were selected and transfected with the FLV and the DVG/FLV ratio was determined over the time. The DVG/FLV ratio at 8h post transfection was plotted against the model (which one?) prediction of P for each variant to demonstrate that higher DVG/FLV ratio implies higher P. Can you say the same for omega?

The full model was used to estimate P from these experimental data on DI mutants. The simplest model that explains the difference between these mutants assumes no difference in encapsidation efficiency. The only nonstructural protein that has been involved in encapsidation is 2C, and none of the mutations we consider are in this specific protein. We added some details in the main text (section 3.4.2, lines 311 and subsequent), and in Supplementary Methods (section ‘Analysis of DI mutants’).

12) What were the criteria for those 8 DVGs to be selected? Have the authors used any bioinformatics methods or tools for variant calling? If so, they should be detailed in the Methods section, too.

We have revised the Method section to described in detail the method used to analyze the sequence of virus populations obtained during co-infection passages (Fig. 5) (lanes 740-768). Positive selected mutations within the DI genome, which frequency increased over passages, were selected and analyzed for replication fitness and competition with WT virus (Fig. 5D and E)

13) Since I am not an experimentalist, it is difficult for me to assess which experiments can and cannot be technically done. Figure 6A and S4 show the impact of different time of delivery of DVGs on the FLV growth. If repeated transfection is possible, experiments to validate these predictions are desirable.

Repeated transfection is not easy technically because second transfection efficiency is usually a lot lower than the first one. To evaluate the impact of different time of delivery of DIs is a future challenge.

14) Lines 460-461: The authors write “This decay might also be real, but we chose not to include it in our model because of the too large number of parameters it has.” It is not acceptable to kill a parameter because it’s inconvenient.

We removed this sentence that does not provide a good justification. The decay of encapsidated genomes is neglected because it is relatively slow, in the scale of days (Walker et al. 2015), compared to the time scale of the experiment, in hours. We have added a sentence to report this (lines 489-491).

Walker, A. T., Williams, A. J., Gary Jr, H. E., Pallansch, M. A., Wassilak, S. G., & Oberste, M. S. (2015). Effect of time at temperature on wild poliovirus titers in stool specimens. *Virology*, *482*, 28-31.

Reviewer #3: Major comments

The idea to use the reduced model to pin down an unidentifiability before going back to the full model is clever, but I suspect the authors could go further with this line of reasoning. I interpret the fact that the reduced model works well (in fact slightly better than the full model) says G_wt(t) + G_di(t) is to an excellent approximation a linear function of R(t), such that dR/dt reduces to the logistic differential equation. Which is to say that the sum of Gs is very fast on the timescale R evolves, or that d(G_wt+G_di)/dt >> dR/dt . I don't quite have it in me tonight to formally work through this slow/fast separation of scales argument, but the authors might do so to better understand why the reduced dynamics should in fact be a good description of the system ‘ (<http://www.bio-physics.at/wiki/index.php?title=Separation_of_Timescales>; also<http://www.scholarpedia.org/article/Multiple_scale_analysis>). From the figures, we can see that the fast dynamics are the competition between G_wt and G_di, such that the total is roughly only a function of R while they play in between. The difference between the singly and dually-infected situations may simply come down to the initial condition (P) and may be derivable by hand (this is supported by the full model results for singly vs dually-infected cells). If this argument holds up, the reduced model may arguably be the better model and the main text can be simplified to only have one model in the figures and with a simpler description of model fitting. (THAT HAVING BEEN SAID, if the authors can't make this argument work, don't find it as enlightening as I do, or in fact I'm just wrong, the current model is publishable.)

We conducted such an analysis, we detailed it in the Supplementary Methods (new section ‘Analytical study of the model’). Thanks to this analysis, we could show that the reduced model is a good approximation of the full model as long as time is less than a given expression (t_0 = 181 minutes). This analysis made us realize that the set of best parameter values that we chose was not the most biologically relevant, as we initially obtained a negative t_0 value. We realized that parameter gamma, the decay rate of cellular resources, was too small, at 7.157x10^-8^ min^-1^. Cellular resources should decay in the range of 1 to 24h, hence gamma should be in the range 6.9x10^-4^ to 1.7x10^-2^ min^-1^. We thus chose another set of best parameter values in this range (see new values in Tab 1, with gamma = 7.471x10^-4^), that indeed yields a positive t_0 value (t_0 = 181 minutes). As a consequence, we had to redo Figures 3 and S2, and changed the fit values for the full model M^123^ in Tab S1. We modified the text accordingly to reflect these changes, that don’t affect the model fit or result interpretation.

Now Fig 5F is a nice prediction!

**Part III – Minor Issues: Editorial and Data Presentation Modifications**

Please use this section for editorial suggestions as well as relatively minor modifications of existing data that would enhance clarity.

Reviewer #1: MINOR POINTS:

a. It is not obvious to this reviewer why it is expected that the mutations in DI will not affect the encapsidation efficiency.

Indeed, based on the design of the genetic selection experiment (Fig. 5B), it is possible that encapsidation can be affected by the mutations selected. However, the majority of the mutations map on the 5’UTR and 3D polymerase which are most likely involved in RNA replication. We thus assumed the simpler possibility. Also following transfection on a single round of replication, we observed an increase on DI replication. Together this data suggest that the DI variants are more effective on RNA replication and unlikely that this is on encapsidation

b. The last sentence of Results is hard to understand.

We have reformulated this sentence and hope it is more clear now.

c. End of Section 4.1: give all units for the range of values for the initial replication rate (is it 3.02-3.07.10E-2 or 3.02.10E-2-3.07.10E-2?)

It is 3.02.10E-2-3.07.10E-2, we have modified the text as suggested.

Reviewer #2: Line 166: limiting resource are produced at a constant rate lambda, not linear, I suppose.

Lambda is a constant linear rate, we changed the text accordingly.

Page 12: Burst sizes are relative or absolute numbers? If the latter, the values are without units (e.g. line 320).

Burst sizes are absolute numbers, we have added the units to the text.

Page 12, line 334: It may be useful to interpret the results with respect to FLV minimization and not FLV maximization or DVG maximization since reduction of FLV should be the goal with DVG interference/treatments.

This paragraph was partially reworded to interpret the results with respect to FLV minimization.

Line 682: The formula for g_wt = g_wt^tot – v_di should probably be g_wt = g_wt^tot – v_wt?

Indeed, we have corrected the formula.

Line 693: It should probably be “…Each optimization consisted in minimizing the sum of squared errors between experimental and simulated normalized data…”. Least squares is a method.

Indeed, this part was corrected as you suggested.

Figure 4, line 6: omega is a ratio not a rate.

Indeed, the legend was corrected.

Supplementary Methods/Description of the models: I’m not sure how the models starting with eq. (13) up to (21) are relevant. The original model is enough as it describes all main determinants of the FLV-DVG dynamics that the authors want to address. And the reduced model is already in the Methods.

We thank the reviewer for the suggestion, but we prefer to keep the models described in Supplementary Methods, because it gives important information on how our model was built based on biological observations. Also, being in Supplement, it does not distract the reader from the model in the main text. Finally, to answer one of the reviews above, we inserted a new Supplementary figure (Fig S1) that shows simulations of model 𝓜^12^ (eqs. S10-S13, omega=1) presented in the Supplementary Methods. Hence it gives one more reason to keep these models' descriptions.

Line 1054: instead of “…minimizing the sum of the least square difference between…” should be “…minimizing the sum of squared differences between…”

Indeed, the method description was corrected.

Reviewer #3: Minor comments

Line 39: I really want an Oxford comma after "apparent"

We added it.

Line 91: I was hoping for an em-dash saying which two steps... (I had to go back to the abstract to find replication and encapsidation, but it would be nice to be re-reminded and cued up for the results after the intro).

We added the reminder and the em-dash.

Figure 3 caption: Is "Experimental data and model predictions" the right phrase? The model was fit to this data and it's not an out-of-sample prediction. I would prefer simply "Experimental data and model" although I acknowledge "prediction" is (over)used for this type of figure by others.

We have replaced ‘model predictions’ by ‘model fit’.

Figure S1E: "-1log" is strange notation. Maybe just "-log"

We have updated the figure.
